# Dr. Balfour in Reply to the Review of His Case of Re-Union of a Wound by the First Intention, after Having Passed into a State of Suppuration

**Published:** 1816-12

**Authors:** 


*For the London Medical and Physical Journal.*

### Dr. Balfour *in Reply to the Review of his Case of Re-union of a Wound by the first Intention, after having passed into a state of Suppuration.*

A FRIEND having put into my hand, the other day, No. 212 of your Journal, containing a critical analysis of my Treatise on Rheumatism, I request those of your readers who are unacquainted with the book, to suspend their judgment of it, till I have time to expose the partial statements, and to repel the illiberal inuendos, of the Reviewer. This I pledge myself to do. I have further to request request of your readers, that, in the mean time, they would have the goodness to compare the *critique* in question with that in No. 197 of your Journal, on the same principles and practice, as first published in the Edinburgh Medical and Surgical Journal, April 1815. Light and darkness are not more inconsistent, than are these critiques with each other.

I take this opportunity of adverting to the observations, in the last Number of your Journal, on my Case of Re-union by the first Intention, published in the Edinburgh Medical and Surgical Journal, October 1816.

The Reviewer observes, "it is absolutely necessary that men should understand one another before they attempt to disprove." To this I most cordially assent; for, had he understood me, he would not have denied that the case in question is an instance of re-union of a wound by the first intention, after having passed into a state of suppuration; nor would he have expressed his opinion in so repulsive a style.

According to the Reviewer, there are *three* ways by which re-union of a wound may take place,—"by inosculation of divided vessels; by coagulable lymph, if the parts are clean, and excited to inflammation; and, by granulations, if they come into contact, *each on the suppurating surface.*" In the northern metropolis, we know of only *two* processes, adhesion and granulation, by which a wound is closed. These, Galen denominated, re-union by the first and second intention.

The Reviewer has omitted to mention the means by which the "inosculation of divided vessels," which, "in the southern metropolis," constitutes "union by the first intent," is *now* effected. In the days of Mr. John Hunter, it was believed to be accomplished through the medium of coagulable lymph and inflammation. So frequently, indeed, is adhesion observed to accompany or follow inflammation, that that great man denominated these processes "adhesive inflammation."

"The process of adhesion or of re-union, by the first intention, is," says Professor Thomson, of the northern metropolis, "through every stage of its progress, accompanied by a greater or less degree of that state which is denominated inflammation."

But the Reviewer himself admits, that, though "the action and configuration of parts is altered, when suppuration has taken place, union may take place by coagulable lymph, if the parts are clean, and excited to inflammation." Now, is not this recurring to the very same phenomena that uniformly formly accompany, or rather constitute, the process of re-union by the first intention? Is not this granting all I contend for, That, in William Cunninghame's case, I have demonstrated that a wound, after having passed into a state of suppuration, will re-unite by the same process which Nature follows in uniting a simple incised wound, without suppuration?

It might have been expected, that, out of regard to candour and truth, the Reviewer would have endeavoured, at least, to refute my arguments before he denied my conclusion. No such thing, however;—this does not suit his taste—dogmatical assertion seems to be his *forte*. Such a mode of proceeding may pass in the "southern metropolis;" it is altogether inadmissible in the "*intellectual city.*"

The Reviewer, in fine, and the subject he attempts to illustrate, are evidently not old acquaintances. His observations are confused and unintelligible; and his conclusion is at variance with his premises.

*Edinburgh; Nov.* 13*th*, 1816.

---

We join with Dr. Balfour in requesting our readers to suspend their judgment concerning our critiques on "Rheumatism," till the author of the papers has spoken in his own defence, only remarking, that the two critiques were written by different gentlemen. But had they been the production of the same writer, we see no reason why further experience should not have altered his opinion.

On the subject of re-union, Dr. Balfour has explained himself more at large. And we can assure him that we have spoken the language of the southern metropolis at this day, and also in the time of Mr. Hunter. That gentleman always described union by coagulating lymph as the adhesive inflammation—a term, we believe, invented by himself. Union by the first intention he conceived the re-union of divided vessels, or, as it is more commonly called, inosculation of divided vessels. This, he remarked, can with difficulty be proved, because, for the most part, the divided surfaces are concealed from our sight. However, as it may be detected in the eye, we are authorized in believing that it exists in other instances of division by a clean cut. See Treatise on the Blood, chap. i. Union by the first intention, page 193, note; and compare this passage with Mr. H.'s account of the "Uniting medium in Inflammations," chap. iii. Union by adhesion, page 307.—EDIT.

